# Supplementary material for: Exploring the clinical utility of rhythmic digital markers for schizophrenia
Source: PLOS Digit Health. 2025 Sep 23;4(9):e0001010. doi: 10.1371/journal.pdig.0001010 (PMC12456810; doi:10.1371/journal.pdig.0001010)
Supplement: S1 Table — Effect sizes (range 0.00-1.00) are Cramer’s V for categorical variables and absolute values of Spearman correlations for continuous variables. Effect sizes ≥ 0.10 (in bold) are considered significant. (DOCX) [file pdig.0001010.s001.docx]

**Supplementary Material**

**S1 Table.** Comparison of participants included and excluded from analyses

|  | **Included   *N=390*** | **Excluded**  ***N=110*** | **Effect sizes** |
| --- | --- | --- | --- |
| Site of recruitment, N (%): |  |  | 0.09 |
| The University of Texas at Dallas | 192 (49.2%) | 42 (38.2%) |  |
| University of Miami | 100 (25.6%) | 36 (32.7%) |  |
| University of California San Diego | 98 (25.1%) | 32 (29.1%) |  |
| Participant group, N (%): |  |  | **0.11** |
| Control | 45 (11.5%) | 9 (8.18%) |  |
| Bipolar disorder | 192 (49.2%) | 43 (39.1%) |  |
| Schizophrenia | 153 (39.2%) | 58 (52.7%) |  |
| Age in years, Median [25th;75th] | 39.0 [31.0;50.0] | 40.5 [29.0;49.0] | 0.05 |
| Gender, N (%): |  |  | 0.05 |
| Men | 156 (40.0%) | 50 (45.5%) |  |
| Women | 233 (59.7%) | 59 (53.6%) |  |
| Other | 1 (0.26%) | 1 (0.91%) |  |
| Ethnoracial groups, N (%): |  |  | **0.18** |
| Asian | 24 (6.15%) | 1 (0.91%) |  |
| Black | 130 (33.3%) | 22 (20.0%) |  |
| Hispanic | 86 (22.1%) | 38 (34.5%) |  |
| Other | 20 (5.13%) | 8 (7.27%) |  |
| White | 130 (33.3%) | 41 (37.3%) |  |
| Educational attainment, N (%): |  |  | 0.09 |
| High school diploma or less | 120 (30.8%) | 39 (35.5%) |  |
| Some college | 134 (34.4%) | 44 (40.0%) |  |
| College degree or higher | 136 (34.9%) | 27 (24.5%) |  |
| Relationship status, N (%): |  |  | 0.04 |
| Not in a relationship | 203 (52.1%) | 62 (56.4%) |  |
| In a relationship | 187 (47.9%) | 48 (43.6%) |  |
| Positive psychotic symptoms, Median [25th;75th] | 13.0 [9.00;17.0] | 15.0 [11.0;19.0] | **0.13** |
| Reduced emotional experience, Median [25th;75th] | 5.00 [3.00;7.00] | 5.00 [3.00;7.00] | 0.02 |
| Reduced emotional expression, Median [25th;75th] | 4.00 [4.00;6.00] | 4.00 [4.00;9.00] | 0.07 |

Effect sizes (range 0.00-1.00) are Cramer’s V for categorical variables and absolute values of Spearman correlations for continuous variables. Effect sizes ≥ 0.10 (in bold) are considered significant.
