# Supplementary material for: Exploring the clinical utility of rhythmic digital markers for schizophrenia
Source: PLOS Digit Health. 2025 Sep 23;4(9):e0001010. doi: 10.1371/journal.pdig.0001010 (PMC12456810; doi:10.1371/journal.pdig.0001010)
Supplement: Attachment — Submitted filename: Response to Reviewers.docx [file pdig.0001010.s004.docx]

Response to reviewers

We thank the reviewers for the excellent comments which greatly helped us in improving the manuscript.

Reviewers’ comments are in *italic*.

Our responses are in **bold**.

Modifications to the manuscript are in green.

Journal Requirements

*2. We noticed that you used “data not shown” in the manuscript. We do not allow these references, as the PLOS data access policy requires that all data be either published with the manuscript or made available in a publicly accessible database. Please amend the supplementary material to include the referenced data or remove the references.*

**We now provide the full results corresponding to the “data not shown” reference (p. 8, lines 373-377).**

Associations were also not significant in analyses restricted to the schizophrenia group (person-level mean of H, mean ratio=1.103; 95% CI: 0.892, 1.364; mean-centered H, mean ratio=0.985; 95% CI: 0.943, 1.028), or in reverse models where H was regressed on person-level mean symptoms (regression coefficient=0.000; 95% CI: -0.010, 0.011) and mean-centered symptoms (regression coefficient=0.001; 95% CI: -0.020, 0.017).

*3. In the online submission form, you indicated that [Data are available from the authors upon reasonable request.*

*All PLOS journals now require all data underlying the findings described in their manuscript to be freely available to other researchers, either
a. In a public repository,
b. Within the manuscript itself, or
c. Uploaded as supplementary information.*

*This policy applies to all data except where public deposition would breach compliance with the protocol approved by your research ethics board. If your data cannot be made publicly available for ethical or legal reasons (e.g., public availability would compromise patient privacy), please explain your reasons by return email and your exemption request will be escalated to the editor for approval. Your exemption request will be handled independently and will not hold up the peer review process, but will need to be resolved should your manuscript be accepted for publication. One of the Editorial team will then be in touch if there are any issues.*

**We added:**

Data are publicly available on the National Institute of Mental Health Data Archive (NDA) website through following the instruction for access and searching for the dataset titled “Introspective Accuracy, Bias, and Everyday Functioning in Severe Mental Illness” (Collection ID: C2941).

**Comments to the Author**

*Reviewer #1*

*This work explore the clinical utility of rhythmic digital markers in schizophrenia. The study is well-founded and well-described, the data and results are clearly presented, the conclusions are fully justified. Data must be made available.*

*Reviewer #2*

*I congratulate the authors for conducting this study. The manuscript contains valuable information and contributes to the expanding body of evidence on digital markers in psychiatry. It offers hope regarding the role of digital technology in enhancing the clinical utility of mental health diagnostics and underscores the potential of digital markers in addressing psychiatric disorders. I have some observations that the authors might consider while revising their manuscript:*

*I recommend abbreviating rhythmic digital markers (RDM) upon its first mention in the body of the article, specifically at line 104. “This study explores the clinical correlates of rhythmic digital markers in schizophrenia...” rather than in line 158.*

**Thank you, we corrected.**

*The structure and flow of the article is somewhat unclear which makes for a difficult read. Why did the authors choose to present their findings before describing the methodology? Additionally, what was the rationale behind placing future directions and limitations ahead of the study’s methodological description?*

**Apologies, we followed the journal requirement for this structure.**

*Again, the placement of the results immediately after the introduction may be confusing for the reader. Can the authors explain why they chose to present their findings before outlining the methodology?*

**Apologies, we followed the journal requirement for this structure.**

*I suggest that the authors include a dedicated conclusion section.*

**We added a conclusion section.**

*All the best!*

*Reviewer #3*

*My main reservation regarding recommending the paper for publication concern the presentation and interpretation of the Markov chains:*

*1a.) 3D plots in Fig. 4 are not needed or useful. The figures are not clear, axes are not labeled, values are strangely scaled. Transition matrices should not be interpolated, they only contain values for prescribed transitions. There are no in-between values.*

**We agree, we removed the 3D representation.**

*1b.) Values in transition cannot be interpreted as attractors or wells they are related to the solutions of the system but they describe dynamics. They do not represent the state space of the system, just the transitions between the states. The attractors/ wells in the system will be probability vectors (with probabilities of being in any of the 9 states after long time). The solutions can also have a form of (probabilistic) cycles of states.*

**We agree, the attractor landscape is the initial state distribution. The matrix is a “transition landscape”. We removed the 3D representation.**

*1c.) The transition matrices are based on a very limited data (for this type of analysis); this might strongly limit reproducibility of the study*

**Thank you for this comment. We add the following in the limitation section of the paper:**

Additionally, self-reports of activities and psychotic symptoms may be affected by desirability and other forms of biases. Finally, our Markov chains were trained on a limited amount of data, given their size (9 states), which might significantly limit reproducibility of the study in the future.

*Some other comments*

*2.) abstract - include n values for each group*

***Corrected***

Data were ecological momentary assessments (EMAs) of 39 activities collected from 390 individuals diagnosed with schizophrenia (N = 153) or bipolar disorder (N = 192) and controls (N = 45)

*3.) line 138 - SMS won't be clear for all audiences use also 'text'*

***Corrected***

*4.) lines 160-170 - consider revising for clarity*

***Corrected***

*5.) line 185 - this statement is in contradiction to the statement in lines 176 and 177 that entropy shouldn't be used; please clarify*

***Thank you, we now clarify as follows:***

Drawing from the idea of social rhythms and dynamic systems theory, we propose to explore the clinical correlates of RDMs based on *time-dependent patterns* of activities entropy and self-reported psychotic symptoms. We use Markov chains to build dynamic indices – the RDMs – out of the fluctuations in those otherwise static indices.

*6.) line 208, equation (1) - missing brackets around p(x)*

***Corrected***

**7.) line 211 - consider revising for clarity, 'Psychologically' isn't typically used in this context. It's also a confusing explanations of entropy.**

**We reformulated:**

A coin flip affords a H of 1 (i.e., full uncertainty), meaning that the level of credence one should have with respect to winning versus losing a bet on a coin flip should be minimal; a maximum entropy corresponds to a minimal level of credence, reflecting the inherent unpredictability of the coin flip. The RDMs we develop in the present study are built out of the Shannon entropy of participants’ social rhythms.

**8.) Lines 217-219 - this explanation is very unclear (it kind of sounds like it says that level of confidence of in wining is 1 when it should be 0).**

***Clarified, we now add***

In eq.1, H(X) represents the H of a random variable X with elements x forming a probability distribution. Formally, H represents the level of uncertainty over X. To make the notion of Shannon entropy (H) more intuitive, it is useful to view it as capturing the level of certainty or credence that an observer should have when sampling X at random. For instance, a distribution made of two elements representing “heads” and “tail” would attribute, for a well-balanced coin, 50% probability to each element of the distribution, or side of the coin. This distribution could be obtained, for instance, by recording the frequencies of heads and tails over, say, a million coin flips. H for that distribution would be 1 (i.e., full uncertainty), which is the maximum entropy for a distribution with 2 elements. This means that the level of credence one should have with respect to winning versus losing a bet on a coin flip should be minimal; indeed, a maximum entropy corresponds to a minimal level of credence, reflecting the inherent unpredictability of the coin flip. The RDMs we develop in the present study are built out of the Shannon entropy of participants’ social rhythms. This means that they track the unpredictability of the activity patterns of participants in each group (e.g., schizophrenia, bipolar, and healthy controls), while reflecting the level of credence one should have over the possibility of finding a participant engaged in one of the 39 possible activities if sampled at random (e.g., how confident a clinician should be that they could predict the activity pattern of their new client vs. in healthy populations).

9.) Figure 1 - Please consider adding an illustration of constructing the activity histograms

**We clarify the caption of figure 1 to highlight how the histogram is constructed:**

**Figure 1. Left hand side.** Each cycle (e.g., C1) is constructed of its cycle entropy (H), which is the entropy of the distribution of activities sampled over 3 days (9 EMA frequencies “|”), and of the mean self-reported symptom score over the 3 days period (SC). The histogram illustrates what H and SC could look like for a hypothetical participant, over 10 cycles (C1-C10). **Right hand side**. The delta in H (grey arrow) and SC (blue arrow) were combined to create the 9 possible states for the transition matrix of the Markov chains.

*10.) line 284 - change 'statement' to 'formula'*

**Changed**

*11.) lines 290-294 - delete; it's enough to say the you used the hittime function*

**Deleted**

*12.) line 306 - example of the distribution should be normalized*

**Corrected**

*13.) line 348 - MEHT is not explained*

**We now add “Mean Expected Hit Times” at first mention**

*14.) lines 362-369 - delete 3d figures are not needed*

**Removed**

*15.) Figure 4. - remove the 3d panels and description in the caption*

**Removed**

*16.) line 401 - consider revising for clarity*

**We clarified:**

Our results provide preliminary evidence of the potential of RDMs as diagnostically informative digital markers.

*17.) lines 404-406 – repetition*

**Corrected**

*18.) lines 412, 416, 425, 430 - there is no analysis showing that discussed differences are significantly shorter/longer* ***±*** *19.) lines 422-424 - I couldn't find analysis that supports that statement; please add reference to a table or figure.*

**These lines correspond to the results in figure 3. We clarify the caption under the result table in figure 3, and add figure 5 that summarises the results, and mention that the interpretation refers to figure 3:**

Group differences in RDMs. Mean H is mean entropy over cycles, MH is the total entropy of the transition matrix, UU, UD, UF, FU, FF, FD, DU, DF, DD are the mean hit times taken by the participants’ Markov chain to reach each of those states (cf. Box 2). Estimates are regression coefficients (uncorrected 95% CI) adjusted for site of recruitment, age, gender, ethnoracial groups, educational attainment, and mean levels of self-reported psychotic symptoms. Significant associations (p<.05) after correction for false discovery rate are indicated in bold. The non-standardized regression coefficient captures the group effect relative to either the control (second column) or the bipolar group (third column) on the variable described on the Y axis, with the negative values reflecting a lower (e.g., faster) value for the variable (e.g., -2.27 reflecting a shorter MEHT to UU in schizophrenia vs. controls), and with the values in bracket below indicating the confidence interval (-4.06, -0.47).

*20.) lines 452-456 - in my view this statement is not supported by the results in the paper*

**We amended the statement and moved it in the future directions section:**

More broadly, one may consider interpreting the dynamic character of illness experience captured by social rhythms dynamics through the lens of lived experience narratives and phenomenological research, by reading (dis)continuities of social rhythms as indicators of shifts towards the emergence or resolution of psychotic experiences. Coupled with phenomenological data, the present approach may be expanded in the future to explore how patterns of insight or realization about the self or the world [[46]](https://paperpile.com/c/4pBZ60/afs8d) follow patterns of variations in social rhythms.

*21.) lines 494-499 - consider revising for clarity*

**We revised to improve clarity and changed the state space language for the state transition language.**

*22.) lines 537-547 - please revise and tone down the statements*

***We remove this section.***
